# Emergency management: ophthalmia neonatorum

**Published:** 2018-11-09

**Authors:** Bolutife Olusanya, Aderonke Baiyeroju

**Affiliations:** 1 Senior Lecturer and Consultant Paediatric Ophthalmologist: Department of Ophthalmology, College of Medicine, University of Ibadan, Ibadan, Nigeria.; 2 Professor and Head of Paediatric Ophthalmology Unit: University of Ibadan, Department of Ophthalmology, College of Medicine, University of Ibadan, Ibadan, Nigeria.

**Ophthalmia neonatorum can cause corneal perforation and intraocular infection.**

**Figure:**
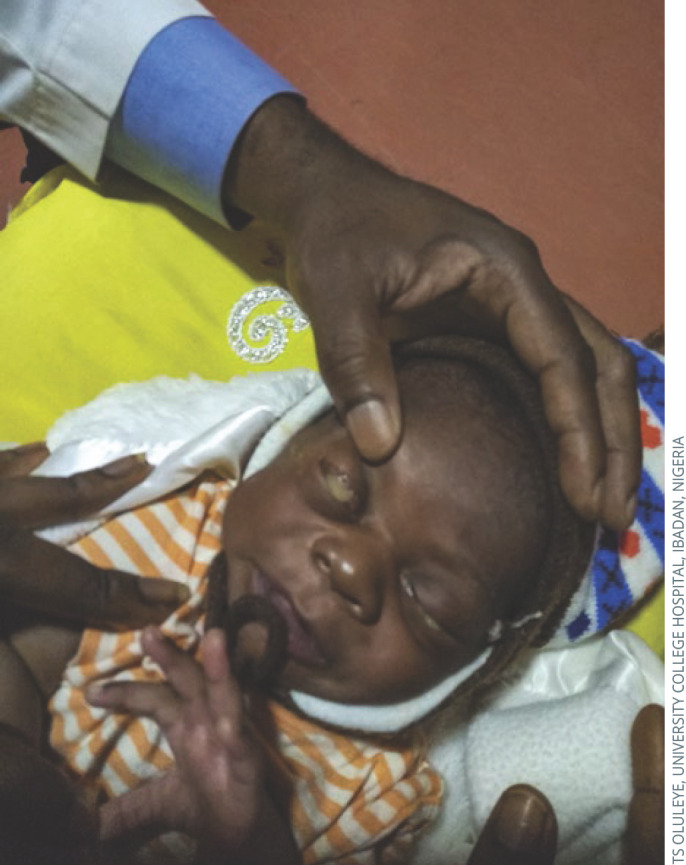
Profuse purulent discharge in a child with ophthalmia neonatorum. NIGERIA

Conjunctivitis in a newborn baby is known as ophthalmia neonatorum (ON). It is an acute emergency and requires immediate treatment and referral because of the significant risk of corneal perforation and intraocular infection that can very quickly lead to blindness.^1^

ON is most common in the babies of mothers infected with the sexually transmitted diseases *Chlamydia trachomatis or Neisseria gonorrhoeae*. Babies' eyes become contaminated during birth.

## Presentation and diagnosis

Ophthalmia neonatorum usually develops between 2 and 14 days after birth. Babies present with redness and swelling of the eyelids, ‘sticky eyes’ and/or discharge from one or both eyes.^2^

## Treatment and referral

Ideally, a swab of the discharge should be obtained in order to determine which organism is responsible. In the absence of easy access to laboratory diagnosis, the World Health Organization recommends that babies should be treated for both gonococcal and chlamydial infections (see panel, right).

For **gonococcal ON**, the recommended treatment is a single dose of intramuscular ceftriaxone injection (50 mg/kg of bodyweight, maximum 125 mg). Alternatives include kanamycin and spectinomycin.^2^

For **chlamydial ON**, the recommendation is 50 mg/kg of erythromycin syrup per day, divided into 4 doses, for 14 days.^2^

Regardless of which organism caused the infection, frequent saline irrigation and cleaning of the eyes is necessary to remove the eye discharge. Topical antibiotics such as erythromycin ointment may be used as an additional therapy. Urgent referral is indicated if there is no improvement within 24–48 hours, or there are signs of sepsis, such as high/low temperature, no interest in feeding, difficulty breathing, vomiting, or if the baby is floppy/unresponsive. In addition, it is important to treat the mother and her partner.

## Prevention

Ophthalmia neonatorum can be prevented before birth by treating maternal infection due to *Chlamydia trachomatis* or *Neisseria gonorrhoea*.

After birth, the infection can be prevented by cleaning the baby's eyes using normal saline and applying an antibiotic eye ointment, such as tetracycline or erythromycin.

Avoid the use of silver nitrate, if possible, as it is associated with chemical conjunctivitis.

**Types of ophthalmia neonatorum**

**Chlamydial conjunctivitis** is the most common type of ON.^1^ It usually presents within 5–14 days of life with redness of the eyes and mucopurulent eye discharge. Most cases are mild to moderate and are self-limited. Eyelid and conjunctival swelling may occur in severe cases. Children with very severe disease may have associated respiratory tract infection.

**Figure 1:**
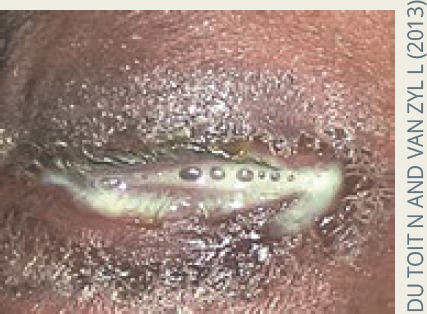
Purulent gonococcal conjuctivitis

**Gonococcal ON** is less common than chlamydial conjunctivitis but is more severe,^1^ so parents are more likely to bring their babies to hospital. It becomes noticeable 2–5 days after birth with copious purulent eye discharge (Figure 1) and severe redness and swelling of the conjunctiva. The eyelids are often very swollen. If untreated, or inadequately treated, it can very quickly result in corneal haziness and perforation, causing blindness. Babies with very severe disease may have systemic complications such as septicaemia and meningitis.

Other conditions that may present with features similar to ON include birth trauma, orbital cellulitis, dacryocystitis and congenital glaucoma. Chemical conjunctivitis may develop as a mild conjunctivitis in a newborn with mucoid eye discharge, redness of the eyes and mild swelling of the eyelids, resolving spontaneously within 48 hours.
